# Factors associated with antihypertensive medication use and blood pressure control in a rural area in Bangladesh: baseline data from a cluster randomised control trial

**DOI:** 10.1186/s12889-021-12379-z

**Published:** 2021-12-23

**Authors:** Fakir M. Amirul Islam, Elisabeth A. Lambert, Sheikh Mohammed Shariful Islam, M. Ariful Islam, Ralph Maddison, Bruce Thompson, Gavin W. Lambert

**Affiliations:** 1 School of Health Sciences, Swinburne University of Technology, Hawthorn, VIC 3122 Australia; 2 Organisation for Rural Community Development (ORCD), Dariapur, Narail Bangladesh; 3 Present Address: Iverson Health Innovation Research Institute, Swinburne University of Technology, Hawthorn, VIC 3122 Australia; 4 Institute for Physical Activity and Nutrition (IPAN), Faculty of Health, Deakin University, Burwood, VIC 3125 Australia

**Keywords:** Hypertension, Antihypertensive medications, Factors associated, Rural Bangladesh

## Abstract

**Background:**

The use of antihypertensive medications is critical for controlling high blood pressure. We aimed to investigate associations between socio-demographic factors and antihypertensive medications use, and antihypertensive medications use with different types of drugs use with levels of systolic blood pressure (SBP) and diastolic blood pressure (DBP).

**Methods:**

For the present report we derived data from the baseline measurements of a cluster randomised control trial on 307 participants with previously diagnosed hypertension from the rural district of Narial in Bangladesh. We measured the participant’s current blood pressure levels and recorded antihypertensive medications uses. Associated factors included socio-economic status, diabetes, antihypertensive medications use, and types of drugs and doses used for controlling blood pressure. We applied analysis of variance and logistic regression techniques to identify factors associated with blood pressure.

**Results:**

Of the total participants, 144 (46.9%) were on antihypertensive medications. After multivariate adjustment, binary logistic regression revealed that employees (odds ratio, (95% confidence interval (CI)) (OR 3.58, 95%CI 1.38-9.28) compared to farmers, and people with diabetes (OR 2.43, 95%CI 1.13-5.26) compared to people without diabetes were associated with a higher proportion of antihypertensive medications use. Of 144 participants on antihypertensive medications, 7 (5%) had taken two doses, 114 (79%) had taken one dose per day and the rest were irregular in medication use. The mean (standard deviation) [min, max] SBP and DBP were 149 (19) mmHg [114, 217] and 90 (10) mmHg [75, 126], respectively. Overall, there was no significant difference in SBP (*p* = 0.10) or DBP (*p* = 0.67) between participants with or without antihypertensive medications or using any type of medications (*p* = 0.54 for SBP and 0.76 for DBP). There was no significant association between antihypertensive medications use and elevated BP levels SBP/DBP≥140/90 mmHg (*p* = 0.42)

**Conclusion:**

Less than half of the people with hypertension were on medication. Irrespective of the antihypertensive medications use, most of the participant’s blood pressure was high. Further study is needed with a large sample to understand the factors and aetiology of unmanaged hypertension in rural areas of Bangladesh where the prevalence of hypertension is very high.

## Background

Hypertension is a major risk factor for disability-adjusted life years and accounts for 19% of deaths or 10.5 million deaths per year worldwide [1–3]. Currently, the estimated prevalence of hypertension is 40% or over 1.1 billion people globally [4]. Lowering blood pressure by 20- mmHg of systolic blood pressure (SBP) or 10-mmHg of diastolic blood pressure (DBP) is associated with a 50% reduction of the risk of a fatal cardiovascular event [5]. Although a significant population with hypertension are unaware of their disease condition, among those with known hypertension, it was estimated that only 18 to 42% of people in high-income countries (HICs) and 8 to 20% in low-income countries were able to control their hypertension [2, 6–10]. Those figures are expected to be disproportionately larger in rural areas where the case fatality due to CVD was also reported to be higher [11].

Previous studies have suggested the prevention and control of hypertension may be improved by the application of targeted and/or population-based strategies. These include interventions to increase awareness in maintaining recommended healthy lifestyle and adherence to antihypertensive medications [10, 12–14], non-adherence to antihypertensive therapy and lifestyle modification are major barriers in managing hypertension [15–17]. Indeed, the prevalence of adherence to antihypertensive medications is estimated to be between 45 and 55% in high-income countries, and 25 to 30% in low-medium income countries. Maintaining medication adherence is a complex issue, especially in patients with chronic diseases where multiple medications and symptoms require attention [18]. Van der Laan et al. [13] noted that non-adherence to antihypertensive medications was associated with a low level of education, low income, younger age, having diabetes, complex medication regimen, multiple dosing regimen, and ethnic variation. Moreover, many patients require aggressive pharmacologic management to control their hypertension [19]. Managing blood pressure at the targeted level is more challenging in low-middle income countries where almost three-quarters of the total hypertensive cases reside, and treatment facilities are insufficient [9].

Bangladesh is a low-middle income country with a large population confronting a significant increase in chronic diseases including hypertension [20, 21]. A systematic review and meta-analyses based on 305,432 participants from 53 studies in Bangladesh reported a wide range of hypertension prevalence, ranging from 1 to 75%. The pooled prevalence was reported to be 41% with a cut-off value of ≥130/80 mmHg and/or use of antihypertensive medications [21]. Islam et al*.* conducted a cross-sectional study among adults aged ≥30 years in a rural area and reported that 40% of adults had hypertension, of which 82% were previously undiagnosed [22] . In a cross-sectional study on people aged 40 years or older in rural communities in Bangladesh, Pakistan and Sri Lanka, Jafar et al. [23] reported that in 53% of people with hypertension in Bangladesh, 71% of people with hypertension in Pakistan and 56% of people with hypertension in Sri Lanka blood pressure remained uncontrolled. Alam et al. [24] conducted a longitudinal study on patients with hypertension and reported that only 37% of rural patients compared to 77% of urban patients visited medically qualified practitioners and that these visits were associated with a mean reduction of 3.3/2.0 (SBP/DBP) mmHg. This indicated that a significant proportion of people with hypertension are unable to control their blood pressure at the targeted level, and that there is a significant gap in service facilities between urban and rural areas. However, the studies had not reported the proportion of patients who were on antihypertensive medications or if the antihypertensive medications were sufficient to control their blood pressure at the targeted level.

The objectives of this study were twofold:

(i) to gain insight into the factors associated with antihypertensive medications use, and
(ii) to determine the current level of blood pressure and examine its association with antihypertensive medication use in a homogenous group of people with diagnosed hypertension.

### Study participants

The study consisted of 307 participants aged 30-75 years recruited for a cluster randomized control trial to estimate blood pressure changes due to lifestyle modification intervention from the Banshgram Union in the Narail District of Bangladesh. The study location is situated approximately 200 km away from the capital city Dhaka.

We selected the study population from the previous cross-sectional study, the Bangladesh Population-based Diabetes and Eye Study, conducted in 2013 in Banshgram among adults aged 30-89 years [22, 25]. In this study, 1242 (40%) participants were identified as having hypertension according to WHO guidelines of SBP ≥140mmHg and /or DBP ≥90 mmHg and or self-report use of medication for hypertension [22]. For the present investigation, given that the intervention program involved lifestyle modification and the use of mobile phones, which is likely more problematic with older participants [26], we limited the age to 75 years, leaving 1072 participants eligible for inclusion in the present study. Recruitment was conducted by local investigators of the Organisation for Rural Community Development (ORCD), a local non-government organization in the Narail district of Bangladesh, along with trained data collectors. The investigators and the data collectors established communication with the potential participants over the telephone or by direct contact. The inclusion criteria for blood pressure was based on recent guidelines and definition of hypertension [27], shifting the cut-off from ≥140mmHg and /or DBP ≥90 mmHg, anyone with SBP ≥130mmHg and /or DBP ≥80 mmHg and or self-report medication for the treatment of hypertension. We excluded people who had advanced CVDs or severe health problems or had not agreed to provide written consent. In the source population of 1072 people, women participation was higher (63%). However, we aimed to recruit approximately equal numbers of women and men. Finally, we recruited 154 men and 153 women for the current cluster RCT. We collected the baseline data for this study from December 2020 to January 2021. We have published the study protocol previously with a detailed description of recruitment strategies [28].

### Baseline data collection and cluster RCT

The ORCD investigators, along with four trained data collectors, obtained data from face-to-face interviews. The data collectors, the local investigator and a physician, participated in four zoom meetings organized by the chief investigator to be trained in data collection. Before the main data collection period, we conducted a pilot study to familiarize the data collectors with all study procedures and allow for an optimal flow of participants. After baseline data collection, we divided the study location into two clusters with 156 participants from cluster 1, approximately 50% of the study location comprising nine villages. The other 151 participants were from cluster 2; the remaining location consisted of the remaining nine villages.

### Statistical power

The current report is based on data dervied from the baseline screening of our RCT, comprising all 307 participants [28]. A previous study in Bangladesh reported that 22% of people in a rural area were on antihypertensive medications [29]. If the proportion of antihypertensive medications use in the current sample would be between 20 to 30%, the sample size was adequate with a statistical power greater than 90% and a significance level of 0.01.

### Outcome variable

The outcome measures were as follows:

1. Primary outcome: Systolic and diastolic blood pressure. We measured blood pressure from the right arm with the person sitting upright and took the second measurement after at least 5 minutes of rest and took the average of the two measures. We took the third measure only if the difference between the first and second measures was more than 20%. In that case, we took the average from the two closest readings. We measured blood pressure using a calibrated Omron Premium Blood Pressure Monitor Device, Omron HEM-7322.
2. Secondary outcome: Use of antihypertensive medications. We asked the participants if they were taking any antihypertensive medications during data collection, which was categorized as “yes” or “no”. If they responded “yes”, data collectors checked their prescriptions to record the types of medicine. Medicines were categorised into five major categories: Amlodipine: Calcium Channel Blocker, Atenolol: Beta-blocker, Amlodipine & Atenolol, Losartan: Angiotensin Receptor Blocker (ARBs), and Amlodipine and Losartan combined. The dose of the medication was recorded, and the frequency was categorised as “once per day”, “twice per day” and “irregular use”.

### Exposure variables

Exposure variables were age, gender, the highest level of education – categorised as no schooling, primary to high school (grade 1 to 9), secondary or higher secondary school certificates (grade 10 to 12 pass), and Bachelor or Master, socio-economic status- categorised as poor, and middle class or rich which was assessed according to Cheng et al. [30]. We categorised the occupation as a housewife, self-managed business, labourers which include digging soils, pulling rikhshaw or any laborious works, and employees who include government and non-government employees. Self-reported use of tobacco categorised as “current smoker” and “non-smoker”, and self-reported diabetes status categorised as “no diabetes”, “with diabetes” and “unknown diabetes” were also used as exposure variables. Secondary outcomes included antihypertensive medications use, doses of medication and medication types were also used as exposure variables in estimating their effects on blood pressure.

### Statistical analysis

We reported the characteristics of participants, including age, level of education and occupation, using descriptive statistics. We used binary logistic regression techniques to estimate the odds ratio (OR) with its 95% confidence intervals (CI) for antihypertensive medication use associated with socio-demographic factors. The reference categories included men for sex, age 30-39 years for age group, and no education level for level of education. The reference groups are shown in the results section. We selected reference categories for categorical variables, assuming that the other categories were on antihypertensive medication at a higher rate than the reference categories. For example, 30-39 years was the reference group for age categories assuming that 40-49 or any older age categories were on antihypertensive medication at a higher rate. Similarly, in the case of SES, poor was the reference category assuming that middle class or rich people were on antihypertensive medication at a higher rate. The lower limit of a 95% CI value greater than or equal to 1.0 for any odds ratio greater than 1.0 indicates significantly higher odds of having an attribute than a factor’s reference group. We presented data as mean with standard deviation (SD) and minimum and maximum value of SBP and DBP by socio-demographic and other factors including antihypertensive medications use, types and dose of medication. We analyzed this using one-way ANOVA. Chi-square test of association was used to report if there was any association between medication use and elevated BP with the cut-off of ≥140/90 mmHg [31]. Statistical software SPSS (SPSS Inc, version 27) was used for the analysis.

## Results

The cohort comprised an almost equal number of men and women, one-third of the participants were over 60 years of age and 15% were between 30- 39 years of age, one-third of the participants had no schooling, half of the participants were homemakers, about one-fifth of the participants were farmers and another one-fifth were employees, one-fifth of the participants were current smokers (Table 1). There was no significant difference in sociodemographic characteristics between the control and the intervention group (Table 2).

**Table 1:** Socio-demographic characteristics, smoking status, diabetes, antihypertensive medications use for the total participants from a cluster RCT in a rural area in Bangladesh

		Number	Percentage
Sex	Female	154	50.2
	Male	153	49.8
Age group, years	Below 40	46	15.0
	40-49	65	21.2
	50-59	95	30.9
	60-69	79	25.7
	70 or older	22	7.2
Level of education	No education	99	32.4
	Primary to high school	148	48.4
	SSC or HSC	43	14.1
	Bachelor or Masters	16	5.2
SES	Poor or very poor	92	30.1
	Middle class	214	69.9
Occupation	Housewife	146	50.5
	Business	24	8.3
	Labour	7	2.4
	Farmer (Agriculture)	59	20.4
	Employees (govt. or private)	53	18.3
Tobacco smoking	Non-smoker	245	79.8
	Current smoker	62	20.2
Diabetes status	No diabetes	217	70.7
	**Diabetes**	41	13.4
	Unknown	49	16.0
Medication use	No medication	163	53.1
	Medication	144	46.9
Medication types^a^	Amlodipine: Calcium channel blocker	32	22.2
	Atenolol: Beta blocker	22	15.3
	Amlodipine & Atenolol	27	18.8
	Losartan: Angiotensin II receptor blockers (ARBs)	23	16.0
	Amlodipine & Losartan	15	10.4
Medication dose^a^	Twice per day	7	4.9
	Once per day	114	79.2
	Irregular	23	16.0

^a^percentage based on the denominator of who are on medication

**Table 2:** Socio-demographic characteristics, smoking status, diabetes, antihypertensive medications use for the total participants and by the intervention status from a cluster RCT in a rural area in Bangladesh

		Total, *N* = 307		Control, *N* = 156		Intervention, *N* = 151		*P*
		Number	%	n	%	n	%	
Sex	Female	154	50.2	74	47.4	80	53.0	0.33
	Male	153	49.8	82	52.6	71	47.0	
Age group, years	30-39	46	15.0	19	12.2	27	17.9	0.19
	40-49	65	21.2	35	22.4	30	19.9	
	50-59	95	30.9	53	34.0	42	27.8	
	60-69	79	25.7	42	26.9	37	24.5	
	70-75	22	7.2	7	4.5	15	9.9	
Level of Education	No education	99	32.4	48	30.8	51	34.0	0.52
	Primary to high school	148	48.4	75	48.1	73	48.7	
	SSC or HSC	43	14.1	22	14.1	21	14.0	
	Bachelor or Masters	16	5.2	11	7.1	5	3.3	
SES	Poor or very poor	92	30.1	51	32.7	41	27.3	0.31
	Middle class	214	69.9	105	67.3	109	72.7	
Occupation	Housewife	146	50.5	41	28.3	31	20.7	0.39
	Business	24	8.3	71	49.0	75	50.0	
	Labour	7	2.4	23	15.9	30	20.0	
	Farmer (Agriculture)	59	20.4	10	6.9	14	9.3	
	Employees	53	18.3	121	77.6	124	82.1	
Tobacco smoking	Non-smoker	245	79.8	35	22.4	27	17.9	0.32
	Current smoker	62	20.2	112	71.8	105	69.5	
Diabetes status	No diabetes	217	70.7	19	12.2	22	14.6	0.83
	Diabetes	41	13.4	25	16.0	24	15.9	
	Unknown	49	16.0	87	55.8	76	50.3	
Medication use	No medication	163	53.1	69	44.2	75	49.7	0.34
	Medication	144	46.9	20	33.3	12	20.3	
Medication types ^a^	Amlodipine: Calcium channel blocker	32	22.2	15	25.0	7	11.9	0.08
	Atenolol: Beta blocker	22	15.3	10	16.7	17	28.8	
	Amlodipine & Atenolol	27	18.8	9	15.0	14	23.7	
	Losartan: Angiotensin II receptor blockers (ARBs)	23	16.0	6	10.0	9	15.3	
	Amlodipine & Losartan	15	10.4	74	47.4	80	53.0	
Medication dose ^a^	Twice per day	7	4.9	4	5.8	3	4.0	0.81
	Once per day	114	79.2	55	79.7	59	78.7	
	Irregular	23	16.0	10	14.5	13	17.3	

^a^percentage based on the denominator of who were on medication

In the total sample, the measure of SBP was mean ± SD [minimum, maximum] 149± 19 [114, 217] mmHg and DBP was 90±10 [75, 126] mmHg. Overall, age (*p* = 0.002 for trend), level of education (*p* = 0.04) and socio-economic status (0.05) were significantly associated with SBP.

The proportion of people on antihypertensive medications was 46.9% (*n* = 144), which was significantly higher in people of age 50-59 years (odds ratio (OR) (95% Confidence Interval (CI): 2.11 (1.01, 4.41)) and 60-69 years (OR (95% CI): 2.88 (1.35, 6.17)) compared to age 30-39 years. After multivariate adjustment, employees (OR (95CI): 4.06 (1.87, 8.83) compared to farmers, and people with diabetes OR (95% CI): 2.14 (1.06, 4.29) compared to people with no diabetes were associated with a higher proportion of antihypertensive medications use. Level of education, socio-economic status or smoking status was not associated with a higher proportion of antihypertensive medications use (Table 3).

**Table 3:** Antihypertensive medications use, and its association with sociodemographic, occupation, smoking and diabetes status using logistic regression from a cluster RCT in a rural area in Bangladesh

		No at risk	N (%)	OR (95% CI) ^a^	OR (95% CI) ^b^
	Total	307	144 (46.9)		
Sex	Male	153	60 (39.2)	1.0 (ref.)	
	Female	154	84 (54.5)	1.86 (0.18, 2.93)	
Age (years)	30-39	46	15 (32.6)	1.0 (ref)	
	40 – 49	65	25 (38.5)	1.29 (0.58, 2.86)	
	50 – 59	95	48 (50.5)	2.11 (1.01, 4.41)	
	60 – 69	79	46 (58.2)	2.88 (1.35, 6.17)	
	70-75	22	10 (45.5)	1.72 (0.61, 4.88)	
Level of education	No education	99	47 (47.5)	1.0 (ref)	1.0 (ref.)
	Primary to high school	148	70 (47.3)	0.99 (0.60, 1.65)	1.35 (0.76,2.42)
	SSC or HSC	43	20 (46.5)	0.96 (0.47, 1.97)	1.77 (0.74,4.2)
	Bachelor or Masters	16	6 (37.5)	0.66 (0.22, 1.97)	1.71 (0.44,6.61)
SES	Poor	92	41 (44.6)	1.0 (ref.)	1.0 (ref.)
	Middle class or rich	214	103 (48.1)	1.15 (0.71, 1.89)	1.24 (0.73,2.12)
Occupation	Farmer	66	17 (25.8)	1.0 (ref.)	1.0 (ref.)
	Housewife	146	80 (54.8)	3.49 (1.84, 6.63)	5.09 (0.86, 30.2)
	Employees	53	31 (58.5)	4.06 (1.87, 8.83)	3.58 (1.38,9.28)
	Business	24	7 (29.2)	1.19 (0.42, 3.35)	1.23 (0.40, 3.84)
Tobacco smoking	Non-smoker	245	117 (47.8)	1.0 (ref.)	1.0 (ref.)
	Current smoker	62	27 (43.5)	0.84 (0.48, 1.48)	1.73 (0.82,3.64)
Diabetes status	No diabetes	217	103 (47.5)	1.0 (ref.)	1.0 (ref.)
	Diabetes	41	27 (65.9)	2.14 (1.06, 4.29)	2.43 (1.13,5.26)
	Unknown	49	14 (28.6)	0.44 (0.23, .87)	0.57 (0.28,1.16)

^a^Odds ratio (OR) (95% confidence interval (CI)): Unadjusted

^b^OR (95% CI) adjusted for the variables in the model except for sex and age

In the total subjects, the measure of SBP was mean ± SD [minimum, maximum] 149± 19 [114, 217] mmHg and DBP was 90±10 [75, 126] mmHg. Overall, age (*p* = 0.002 for trend), level of education (*p* = 0.04) and socio-economic status (0.05) were significantly associated with higher SBP. Participants of age 30- 39 years had significantly lower SBP 141±12 [123, 178] compared to those 50 years or older, such as for 50-59 years of age SBP was 149±19 [114, 205]. No education 152±17 [125, 208] compared to any levels of education, and being poor 150±20 [114, 205] compared to the middle class or above were associated with significantly higher mean SBP. Tobacco smoking was not associated with either SBP or DBP. None of the factors was associated with DBP.

There was no difference in between SBP or DBP in those taking or not taking antihypertensive medications. Medication type and the dose taken was not associated with SBP or DBP (Table 4).

**Table 4:** Blood pressure as the outcome variable by socio-demographic, behavioural factors, antihypertensive medications use, and dose of medication as exposure using one-way ANOVA from a cluster RCT in a rural area in Bangladesh

	Total participants				
		Systolic blood pressure (SBP)		Diastolic blood pressure (DBP)	
	No at risk	Mean ± SD [Min, Max]	P*	Mean ± SD [Min, Max]	P*
Total	307	(149±19) [114, 217]		(90±10) [75, 126]	
Sex					
Female	154	(150±17) [116, 205]	0.08	(90±9) [75, 118]	0.54
Male	153	(148±20) [114, 217]		(89±10) [75, 126]	
**Age (years)					
30-39^a^	46	(141±12) [123, 178]	0.002	(90±8) [78, 118]	0.87
40 - 49 ^a^	65	(147±15) [128, 210]		(91±9) [76, 126]	
50 - 59 ^b^	95	(149±19) [114, 205]		(90±9) [75, 117]	
60 - 69 ^b^	79	(152±21) [114, 217]		(89±11) [75, 126]	
70-75 ^b^	22	(158±22) [128, 208]		(89±11) [75, 118]	
**Education					
No education ^a^	99	(152±17) [125, 208]	0.04	(90±9) [75, 118]	0.96
Primary to high school ^a^	148	(147±19) [114, 217]		(90±10) [75, 126]	
SSC or HSC ^a^	43	(150±18) [123, 198]		(90±9) [77, 126]	
Bachelor or Masters ^b^	16	(140±16) [130, 196]		(89±8) [75, 105]	
SES					
Poor	92	(150±20) [114, 205]	0.05	(90±10) [76, 118]	0.12
Middle class	214	(148±18) [114, 217]		(90±10) [75, 126]	
Occupation					
Housewife	146	(150±17) [116, 205]	0.43	(90±9) [75, 118]	0.48
Business	24	(149±20) [129, 210]		(92±12) [77, 126]	
Labour	7	(142±9) [131, 154]		(89±8) [78, 98]	
Farmer	59	(146±19) [114, 217]		(88±10) [75, 121]	
Employees	53	(150±21) [114, 208]		(89±10) [75, 118]	
Tobacco smoking					
Non-smoker	245	(149±18) [116, 217]	0.34	(90±10) [75, 126]	0.66
Current smoker	62	(147±20) [114, 210]		(89±10) [75, 126]	
Diabetes status					
No diabetes	217	(149±20) [114, 217]		(90±10) [75, 126]	0.43
Diabetes	41	(151±18) [125, 208]		(89±10) [75, 118]	
Unknown	49	(146±14) [121, 191]		(89±9) [75, 113]	
Medication use					
No medication	163	(147±16) [123, 198]	0.10	(90±9) [75, 126]	0.67
Antihypertensive medications	144	(151±21) [114, 217]		(90±10) [75, 126]	
Medication type					
Amlodipine: Calcium channel blocker	32	(147±18) [124, 205]	0.54	(90±7) [75, 105]	0.76
Atenolol: Beta blocker	22	(147±15) [129, 177]		(90±6) [80, 101]	
Amlodipine & Atenolol	27	(153±19) [130, 210]		(91±13) [75, 126]	
Losartan: Receptor blocker (ARBs)	23	(152±23) [116, 200]		(88±10) [75, 115]	
Amlodipine & Losartan	15	(144±25) [114, 208]		(92±14) [76, 118]	
Medication dose					
Twice per day	7	(150±28) [114, 193]	0.53	(96±15) [76, 118]	0.28
Once per day	114	(150±20) [116, 210]		(90±10) [75, 126]	
Irregular	23	(155±25) [114, 217]		(91±10) [79, 121]	

*P indicates significance levels

** the same letter indicates no significant difference

Of participants who were on medication, 73% had elevated BP (≥140/90 mmHg) compared to 69% who were not on antihypertensive medication (*p* = 0.42). Although there was no significant association between types of medication and blood pressure, atenolol was associated with the highest proportion of individuals with elevated BP (86.4%), and the combination of amlodipine & losartan had the lowest proportion with elevated BP (46.7%) (*p* = 0.10) (Table 5).

**Table 5:** Association of antihypertensive medication use, medication type and dose with people who were able to manage their blood pressure at <140/90 mmHg level using Chi-square tests

	<140/90 mmHg level, number (%)	≥140/90 mmHg level, number (%)	P*
Total	90 (29.3)	217 (70.7)	
**Medication status**			
No antihypertensive medication	51 (31.0)	112 (69.0)	0.42
Antihypertensive medications	39 (27.0)	105 (73.0)	
**Antihypertensive medications types**			
Amlodipine: Calcium channel blocker	10 (31.3)	22 (68.8)	0.10
Atenolol: Beta blocker	3 (13.6)	19 (86.4)	
Amlodipine & Atenolol	6 (22.2)	21 (77.8)	
Losartan: Receptor blocker (ARBs)	6 (26.1)	17 (73.9)	
Amlodipine & Losartan	8 (53.3)	7 (46.7)	
**Medication dose**			
Twice per day	2 (28.6)	5 (71.4)	0.99
Once per day	31 (27.2)	83 (72.8)	
Irregular	6 (26.1)	17 (73.9)	

*p for associations between BP status and medication and dose use

## Discussion

In this study we report the prevalence of antihypertensive medication use, doses of medication and their association with current blood pressure level in people with previously diagnosed hypertension in a typical rural area in Bangladesh where the prevalence of hypertension has been shown to be 41% [32]. The significant findings from this study include: (1) that less than half of the participants previously diagnosed with hypertension were currently on medication, (2) employees and patients with diabetes were more likely to be on medication, (3) higher education and younger age was associated with lower level of systolic blood pressure (4) almost two-thirds of the participants had blood pressure ≥140/90 mmHg SBP/DBP, and (5) the use of medication was not shown to be associated with achieving BP level <140/90 mmHg.

In the current study, the association of higher education and younger age with a lower level of blood pressure is consistent with previous studies [33–35]. Association of higher education with lower level of blood pressure may be due to educated people being more aware of managing health and adhere to regularity in treatment follow-up or taking regular physical exercise [34], and that they can afford medications compared to people with a low level of education who work in lower paying employment. This is supported by our findings that employees were significantly associated with a higher proportion of taking medication than those who worked in lower-paying employment, higher education was also shown to have an upward trend in taking antihypertensive medications. Vargas et al. [35] have reported that people with education level 12 years or higher among non-Hispanic Whites who were younger than 25-44 years of age was associated with a lower incidence of hypertension both in men and women compared to those with a lower level of education. We have found that there was an association between being older and a higher level of blood pressure. Higher level of blood pressure among older people can be partly because BP is more difficult to manage among this vulnerable group, and this is consistent with a previous finding [36].

In the present study we relied on self-reported medication use by checking of medication prescriptions. Our findings are consistent with previous studies which reported that non-adherence to antihypertensive medication to occur between 30 and 50% [33, 34]. A study of self-reported medication use compared with pharmacy records, especially for statins use, calcium channel blockers, β-blockers, and bisphosphonates among older women [37], showed almost perfect agreement. In terms of medication taken, a multicentre study of hypertension, its awareness, treatment and control among older adults in India and Bangladesh [29] reported that 389% of participants had taken antihypertensive treatment in urban, and 22% in rural settings. In our study, 44% of participants were on medication and 73% of participants had elevated blood pressure, as indicated by clinic BP ≥140/90 mmHg [31], which is broadly consistent with studies in other low-middle income countries, such as was reported in the PURE study [38], which reported elevated blood pressure was 52.8% in Bangladesh, 70.6% in Pakistan and 56.5% in Sri Lanka [23]. Another multicentred study in India and Bangladesh reported that among treated patients, 31.9% had elevated BP in the urban setting and 53.6% had elevated BP in the rural setting [29].

Previous studies have demonstrated a clear association between blood pressure control and lifestyle factors, including diet and exercise, ageing, obesity, family history, low salt intake and physical activity [39–43]. Activation of the sympathetic nervous system and the renin-angiotensin-aldosterone system have been implicated in initiating and sustaining blood pressure elevation and are targets for therapy [44]. Indeed, anti-hypertensive medication [45, 46] has consistently been associated with lowering blood pressure. Our study reported that there was no difference in managing blood pressure in people who were and who were not on medications. This could be due to a reason of non-adherence to antihypertensive medications, which is one of the most important factors of uncontrolled blood pressure [13]. In our study, information on medication use was self-report and we did not have any measure of participants actually taking their medications, such as visual inspection or urine analysis of drug metabolites. A study in Germany provided some evidence that increased uptake of polytherapy has increased the BP control rate from 42 to 72% in a decade [46]. However, in our study, less than 50% of the participants who were on medications, three-quarters of these took one dose, 5% of them had taken 2 medications per day and the rest were irregular. This result of taking one dose is consistent with a previous finding conducted in South Asia which reported that 67% of patients took one medication and this was not associated with controlled blood pressure [23]. In the SPRINT study, it was reported that patients required one additional medication to reach the targeted BP compared to those who had uncontrolled BP in the treated group [47]. In the current study, one-third of the people younger than 40 years were on medication compared to more than 50% of those aged 60 years or older, and the mean blood pressure was significantly higher among the older people. This indicates, in older age or people with elevated BP, more aggressive pharmacologic management or personalised patient care may be required to control BP at the targeted level [19]. In our study, although there was no significant association between types of medication use and elevated BP levels, atenolol was associated with the highest proportion of individuals with elevated BP and amlodipine and losartan combined with the lowest proportion of elevated BP which is consistent with the findings that at least two antihypertensive drugs are required for most hypertensive patients to achieve BP at targeted level [48].

National guidelines for the management of hypertension in Bangladesh were prepared in 2013 [49]. The policy defined hypertension with a blood pressure cut-off of 140/90 mmHg or higher. Several recommendations were made for managing hypertension, such as maintaining BMI between 18-5-23.5 kg/m^2^, consumption of salt intake <5 gm/day and undertaking moderate-intensity physical activity for at least 30 minutes on most days. Initiation of pharmacotherapy was advised for individuals with stage 1 hypertension, with follow up and use of additional classes of medications for those with higher pressures or if management to target pressure was not achieved. Our study indicated that half of the people with elevated BP were on medication and took only one dose. There are several barriers to lowering BP at an individual and community level, such as having a low level of education, reduced health literacy, low income and, in some jurisdiction, female gender, especially in low-middle income countries [13]. Health inequities may be more evident in rural areas where healthcare facilities, adequate infrastructure, availability of trained clinicians are very different compared to the urban setting [50].

Our study has several strengths: Firstly, the face-to-face data collection from a sample based on their previous participation in a cross-sectional study. The sample consists of almost 50% of women and from a homogenous group across different occupations, age and level of education. However, the study is not free from limitations. Firstly, the recruitment is from a cluster RCT which was not necessary to have a large sample, and thus the sample size limits many sub-analyses and, in some cases, failed to show significant p values although the effect sizes are large. Secondly, the study was conducted in one rural location. Therefore, the results cannot be generalised at the national level. However, the rural population and the health system are very similar in Bangladesh. Finally, medication data was based on self-report and the medication package check and not based on more precise indicators such as visual observation of medications being taken or analysis of urinary medication metabolites.

## Conclusions

Our study has provided information on antihypertensive medication use and its association with blood pressure levels in rural Bangladesh. Less than half of the previously diagnosed participants with hypertension were currently on antihypertensive medications. Of people who are on medication, three-quarters took one medication. Almost three-quarters of the participants with previously diagnosed hypertension have elevated BP irrespective of whether they were on medication or not.

## Data Availability

The datasets used and/or analysed during the current study are available from the corresponding author on reasonable request.

## Abbreviations

ORCD: Organisation for Rural Community Development
SBP: Systolic Blood Pressure
DBP: Diastolic Blood Pressure
CI: Confidence Interval
CVD: Cardiovascular Disease
OR: Odds Ratio
ANOVA: Analysis of variance
